# β-Hydroxybutyrate Effects on Bovine Caruncular Epithelial Cells: A Model for Investigating the Peri-Implantation Period Disruption in Ketotic Dairy Cows

**DOI:** 10.3390/ani13182950

**Published:** 2023-09-18

**Authors:** Carolin Hildebrand, Julia Hollenbach, Bettina Seeger, Christiane Pfarrer

**Affiliations:** 1 Institute of Anatomy, University of Veterinary Medicine Hannover Foundation, Bischofsholer Damm 15, 30173 Hanover, Germany; carolin.anja.hildebrand@tiho-hannover.de (C.H.); julia.hollenbach@tiho-hannover.de (J.H.); 2 Research Group Food Toxicology and Alternative/Complementary Methods to Animal Experiments, Institute for Food Quality and Safety, University of Veterinary Medicine Hannover Foundation, Bischofsholer Damm 15, 30173 Hannover, Germany; bettina.seeger@tiho-hannover.de

**Keywords:** ketosis, high-yielding dairy cows, β-Hydroxybutyrate, bovine caruncular epithelial cells, cell culture model, peri-implantation period

## Abstract

**Simple Summary:**

In high-yielding dairy cows, the successful establishment of pregnancy is of great economic importance. If a new pregnancy does not occur shortly after parturition, this can mean early culling for the animal. Due to today’s breeding, cattle show higher milk yields, but pregnancy rates continue to decline. In addition to genetic dispositions, metabolic diseases, such as ketosis, are particularly relevant here. In this in vitro study, bovine uterine cells were stimulated with the main ketone body β-Hydroxybutyrate. The influence of β-Hydroxybutyrate on uterine cell function, inflammatory state and important genes for successful implantation was investigated. These genes include cell adhesion molecules and prostaglandins. High concentrations of β-Hydroxybutyrate negatively affected cell function and induced an inflammatory response in the cells. These results provide a possible explanation for the poor reproductive performance of ketotic dairy cows. Due to the reduced cell function as well as inflammatory response, the finely regulated feto-maternal communication during early pregnancy might be disturbed.

**Abstract:**

Ketosis is a metabolic disorder arising from a negative energy balance (NEB). It is characterized by high β-Hydroxybutyrate (BHBA) blood levels and associated with reduced fertility in dairy cows. To investigate the impact of BHBA on bovine caruncular epithelial cells (BCEC) in vitro, these cells were stimulated with different concentrations of BHBA. Cell metabolism and motility were examined using an MTT assay and Live-cell imaging. RT-qPCR was used to examine mRNA expressions of *TNF*, *IL6*, *RELA*, prostaglandin E2 synthase (*PTGES2*) and receptor (*PTGER2*) as well as integrin subunits *ITGAV*, *ITGA6*, *ITGB1* and *ITGB3*. Stimulation with 1.8 and 2.4 mM of BHBA negatively affected cell metabolism and motility. *TNF* showed increased mRNA expression related to rising BHBA concentrations. *IL6*, *RELA*, *ITGAV*, *ITGA6*, *ITGB1* and *ITGB3* as well as *PTGER2* showed no changes in mRNA expression. Stimulation with 0.6 and 1.2 mM of BHBA significantly increased the mRNA expression of *PTGES2*. This does not indicate a negative effect on reproductive performance because low BHBA concentrations are found in steady-state conditions. However, the results of the study show negative effects of high BHBA concentrations on the function of BCECs as well as an inflammatory response. This could negatively affect the feto-maternal communication during the peri-implantation period in ketotic dairy cows.

## 1. Introduction

In high-yielding dairy cows, a negative energy balance (NEB) often appears during the transition period, spanning from three weeks pre calving until three weeks post calving [1]. Late gestation, parturition, and the onset of lactogenesis require a high amount of energy, with a simultaneous reduction in feed intake [2,3]. During this time, many metabolic adaptions are required, and genetic dispositions as well as environmental factors and infections can lead to a metabolic imbalance [4]. A very common disease resulting from maladaptation to NEB is ketosis [3,5]. It is characterized by increased blood levels of ketone bodies, whereas β-Hydroxybutyrate (BHBA) is the most frequent one [6,7,8]. BHBA is synthesized from acetyl-CoA in the mitochondria of the liver during high rates of lipolysis [9]. Depending on the literature, the threshold value of BHBA blood concentration for ketosis is between >1.2 mM and ≥1.4 mM [7,8,10]. Cattle affected by NEB and elevated BHBA blood concentrations show an increased prevalence of mastitis, lameness, metritis, reduced milk yield and poor reproductive performance [11,12,13,14].

BHBA is a versatile molecule [15,16]. In the organism, it can be used as an alternative energy source by many tissues (reviewed by [15,16]). For cells, various effects of BHBA are described in the literature. On the one hand, BHBA promotes cell viability and cell metabolism, and on the other hand, it promotes the expression of anti-proliferative and proapoptotic genes via inhibition of histone deacetylation [16,17,18,19,20]. This mechanism is also called the “butyrate paradoxon” (reviewed by [21]), which, after Donohoe et al. [20], can be explained by the “Warburg effect”. In cancer cells that obtain their energy mainly by aerobic glycolysis and not by oxidative metabolism, BHBA is metabolized inefficiently and accumulates in the nucleus. This results in an increased inhibition of histone deacetylation and may inhibit cell proliferation. In contrast, BHBA has a growth-promoting effect when used as an oxidative energy source in normal cells [20]. Furthermore, in bovine hepatocytes and endometrial cells, BHBA causes an inflammatory response by inducing oxidative stress and activation of the nuclear factor-kappa B (NF-κB) signalling pathway in vitro. The expression of cytokines such as interleukin-6 (*IL6*) and tumour necrosis factor α (*TNF*) is increased in these cells [22,23]. In addition, in vivo studies also observed an inflammatory response in ketotic cows. Affected animals show elevated blood levels of proinflammatory factors and increased expression of Toll-like receptors 2 and 4 as well as increased phosphorylation levels of NF-kb p65 in neutrophil granulocytes [24,25,26,27]. At the same time, the likelihood of pregnancy is significantly reduced in cows diagnosed with ketosis compared to healthy animals [11].

Although poor reproductive performance in cows suffering from ketosis may affect all stages of the reproductive cycle, early pregnancy is a well-known critical period [28]. It is defined as the period until day 28 post insemination and is characterized by embryonic maturation from the blastocyst through the elongation stage up to implantation [28,29]. During this time, 70–80% of the abortions take place [30], and the main reasons are genetic abnormalities, premature luteolysis and asynchronous feto-maternal communication [31,32,33]. In cows, the *semiplacenta multiplex seu cotyledonaria* consists of fetal cotyledons interdigitating with maternal caruncles. The caruncles are located in the lumen, coated by caruncular epithelial cells and represent the glandless surface for feto-maternal exchange [34]. For successful pregnancy establishment, a precise regulation of gene expression in cells involved in the implantation process is of great importance. The cytokine Interferon-τ is the pregnancy recognition signal of the ruminants [35], but also, other cytokines like IL6, IL1 and the leukemia inhibitory factor (LIF) are involved in the implantation process of mammals and promote embryonic development [36]. In addition to cytokines, the prostaglandin F2α (PGF2α) and prostaglandin E2 (PGE2) are important regulators during early pregnancy. While PGF2α activates luteolysis, PGE2 promotes the secretion of gonadotropins, which support the formation and maintenance of the corpus luteum [37,38,39]. Furthermore, PGE2 supports the hatching of the blastocyst from the *zona pellucida*, and it is involved in ovulation and early implantation [40,41]. Besides playing an important role in reproductive mechanisms, prostaglandins are also involved in inflammatory processes [42].

Other key players during implantation are transmembrane proteins called integrins. Through their cell-to-cell as well as cell-to-extracellular matrix binding, they act as mediators of cell communication and are important for the maintenance of physiological cell functions [43]. In sheep, which like cattle have a synepitheliochorial placenta, the integrin α_v_β_3_ plays a crucial role in implantation. Here, the progesterone-mediated extracellular matrix protein osteopontin acts as a binding partner for the integrin α_v_β_3_ and promotes the adhesion and migration of the trophoblast to the uterine luminal epithelium [44]. In other species, the integrin subunits α_v_ and β_3_ are important requirements for implantation in the endometrium [45,46,47]. In cattle, these integrin subunits were also detected at the endometrium [48]. A cycle-dependent and implantation-associated change in the expression pattern of integrins was also observed in the bovine uterus [49,50]. During the implantation window, mainly the subunits α_1_, α_3_ and α_6_ are expressed [50]. In a study of trophoblast giant cell migration, the feto-maternal connection is assumed to be mediated through laminin and the integrin α_6_β_1_ [48]. Another in vitro study on bovine endometrial cells pointed out that laminin and the integrin α_6_β_1_ are expressed in placentomes of cattle [51].

The hypothesis of the present study is that BHBA, as a marker of ketosis, disrupts bovine caruncular epithelial cell (BCEC) physiology and gene expression patterns, and thereby, impairs the sensitively regulated peri-implantation period. To test this hypothesis, BCECs were stimulated with different concentrations of BHBA, and the viability of the cells was examined by analysing motility and metabolism. Furthermore, the influence of BHBA on the mRNA expression of inflammatory markers (*IL6*, *TNF* and *RELA* (gene encoding for NF-κB p65)), the prostaglandin E2 synthase (*PTGES2*) and receptor (*PTGER2*) and different integrin subunits (*ITGAV*, *ITGA6*, *ITGB1* and *ITGB3*) was examined. The results may elucidate pathomechanisms to explain poor reproductive performance associated with NEB in high-yielding dairy cows.

## 2. Materials and Methods

### 2.1. Cell Culture

The established and characterized BCEC line [52] was used in the study. BCECs were isolated from maternal placentomes of a pregnant cow [52]. Cells were stored in cryotubes at −150 °C at the Institute for Anatomy, University of Veterinary Medicine Hannover, Foundation, Germany. For cultivation, cells were thawed and seeded into T75 flasks (TPP Techno Plastic Products AG, Transadingen, Switzerland) filled with Dulbecco’s Modified Eagle Medium (DMEM)/Ham’s F12 (Sigma-Aldrich^®^ Chemie GmbH, Taufkirchen, Germany). DMEM was supplemented with 10% fetal calf serum (FCS) (Biochrom, Berlin, Germany), Penicillin (100 µU/mL, PAA, Coelbe, Germany), Streptomycin (2 µg/mL, PAA, Coelbe, Germany) and L-Glutamine (2 mM, PAA, Coelbe, Germany). This culture medium will be called full medium (FM) in the following. Cells were cultivated in an incubator at 5% CO_2_ and 37 °C, and culture medium was replaced every other day. Subculturing was performed at 90% confluency via trypsinisation for 5 min at 5% CO_2_ and 37 °C using 0.5% Trypsin (Sigma-Aldrich^®^ Chemie GmbH, Taufkirchen, Germany) in PEM (EDTA 2 mM in phosphate-buffered saline (PBS)). For the experiments, cells were prepared individually. More detailed information will follow in the respective chapters. Cells between cell passage 18 and 32 were used in the experiments.

### 2.2. Stimulation of BCECs with BHBA

Stimulation was started when the cells reached a confluence of about 70%. FM was replaced by serum-reduced medium (SR), with 1% FCS and the same amount of Penicillin, Streptomycin and L-Glutamine added to the DMEM, as in FM, and incubated for 3 h before stimulation with different BHBA concentrations. To prepare a 20 mM BHBA stock solution, 63.045 mg of BHBA powder (Sigma-Aldrich^®^ Chemie GmbH, Taufkirchen, Germany) was dissolved in 25 mL of distilled water. Subsequently, the stock solution was sterile filtered and stored at −20 °C. For the stimulation experiments, the stock solution was diluted in SR. The following concentrations were used: 0.6, 1.2, 1.8 and 2.4 mM of BHBA and stimulations were carried out for 24 or 36 h (cell metabolism).

### 2.3. Cell Metabolism (MTT Assay)

Cell metabolism was examined via MTT assay according to Mossman [53]. In this process, a water-soluble yellow dye is converted by the cells into violet crystals, the formazan salt. These crystals cannot pass the cell membrane and stay intracellular. BCECs were seeded into a 96-well plate (TPP Techno Plastic Products AG, Transadingen, Switzerland) at a cell number of 1 × 10^4^ cells/well and cultured in FM for 24 h. After stimulation of BCECs for 24 and 36 h, 5 mg of MTT (3-(4,5-dimethylthiazol-2-yl)-2.5-diphenyltetrazoliumbromid; Carl Roth GmbH, Karlsruhe, Germany)-reagent was dissolved in 5 mL of PBS. This solution was diluted 1:10 in SR and applied to the cells. After 2.5 h of incubation, MTT-reagent was removed and Dimethylsulfoxide (DMSO, Carl Roth GmbH, Karlsruhe, Germany) was added to the BCECs. Subsequently, the optical density was determined using an ELISA-Reader (Thermo Fisher Scientific, Waltham, MA, USA). The extinction was measured at 550 nm (sample absorbance) and 690 nm (background absorbance). The experiment was repeated fourteen times, and for each stimulation group, between 177 and 199 values were captured, whereby values of wells were eradicated if BCECs detached during stimulation. During the MTT run for 36 h, between 181 and 198 values were collected for each stimulation group.

### 2.4. Cell Motility (Live-Cell Imaging)

Cell motility was examined via Live-cell imaging. For this purpose, 8 × 10^4^ cells/well were seeded into a 12-well plate (Sarstedt AG& Co. KG, Nürnbrecht, Germany) and cultured in FM for 24 h. After cultivation, BHBA was added to the BCECs, and the cells were transferred to the incubation chamber of a Cell Observer System (Zeiss, Jena, Germany). A picture of the BCECs was taken every 20 min at two representative areas of each well during the 24 h of stimulation period. The analysis of the accumulated distance was carried out through manual tracking of 20 cells per determined area using the chemotaxis tool of the ImageJ program [54]. The experiment was repeated four times, and for each stimulation group, 320 distances were determined.

### 2.5. RNA Isolation, cDNA Synthesis and Quantitative Real-Time PCR

For the BHBA stimulation, 5.5 × 10^5^ cells/mL were seeded in 6 mm cell culture dishes (TPP Techno Plastic Products AG, Transadingen, Switzerland) and grew to a confluence of 60–70%. After stimulation with BHBA, cells were harvested, and RNA was extracted using the NucleoSpin^®^ RNA and Protein purification kit (Macherey-Nagel GmbH & Co., KG, Düren, Germany) according to the supplier’s manual. The RNA concentration was analysed by determining the optical density with a spectrophotometer (DeNovix^®^ DS-11 Spectrophotometer, DeNovix Inc., Wilmington, NC, USA) at 260 nm. Additionally, the purity of the RNA samples was determined by the absorption quotients of 260/280 nm and 260/230 nm. Only samples with an ideal ratio at 2.0–2.2, respectively [55], were used in the further experiments. For cDNA synthesis, the GoScript™ Reverse Transcriptase System (Promega GmbH, Mannheim, Germany) was used according to the manufacture’s introductions. For a cDNA concentration of 50 ng/µL, 1 µg of total RNA in a 20 µL reaction volume was used. The success of cDNA synthesis was confirmed with a conventional standard PCR using primers of the reference genes *ACTB* or *GAPDH*. Afterwards, the expression levels of the mRNA were quantified using quantitative Real-Time PCR (RT-qPCR). Primers were designed with the PrimerBLAST software from the National Center for Biotechnology Information (NCBI) (accessed on 3 and 19 January 2022) and purchased from Microsynth AG (Balgach, Switzerland) or are already published in the previous literature. Before use in RT-qPCR, the most appropriate master mix for each primer pair was chosen. The different master mix compositions are shown in Table 1. Afterwards, the efficiency of each primer pair was determined using the standard curve method with seven dilution steps of a 1:2 dilution series. Only primers with an amplification efficiency between 90–110% were used in the RT-qPCR. Detailed information of the primers is shown in Table 2. For the preparation of the reaction mix, cDNA was diluted with nuclease-free water to a final concentration of 5 ng/µL, and 4 µL of the cDNA was mixed with the reaction components containing SYBR^®^ Green as the fluorescent dye (Applied biosystems, Waltham, MA, USA) (Table 1). For each primer pair, a negative control was implemented, containing nuclease-free water instead of cDNA. A Stratagene Mx3000P (Aglient Technologies, Waldbronn, Germany) RT-qPCR cycler with the following cycling program was used: first, it was heated to 95 °C for polymerase activation; second, there were 40 cycles for amplification, whereby each cycle consisted of 15 s of 95 °C (denaturation) and 60 s of 60 °C (annealing and extension), with fluorescence detection during the annealing and extension step. Subsequently, a melting curve analysis consisting of 15 s of 95 °C, a 60 min temperature increase up to 95 °C in 0.3 °C steps and lastly holding for 15 min at 95 °C were conducted. Each stimulation experiment was repeated six times with all samples analysed in duplicates. The ΔΔCt method was used for relative mRNA quantification using *ACTB* and *GAPDH* as reference genes and the control group SR as the untreated sample.

### 2.6. Statistical Analysis

Statistical analysis was performed using “The R Project for Statistical Computing” (version 4.2.0). For the statistical analysis, the arithmetic mean values and standard deviations were first determined using descriptive statistics. Subsequently, the Shapiro–Wilk-test was performed to test whether the data were normally distributed. If the data were not normally distributed, the non-parametric Kruskall–Wallis test was used. In case of significant results, the Dunn test with Bonferroni correction was performed as a post hoc test. For normally distributed data, a one-way ANOVA was used, followed by Tukey’s HSD test with Bonferroni correction. In cases of inhomogeneous variance, the Games–Howell test was used instead. The level of significance was set at 5%.

## 3. Results

### 3.1. Effects of BHBA on the Metabolism and Motility of the BCEC

**Figure 1:**
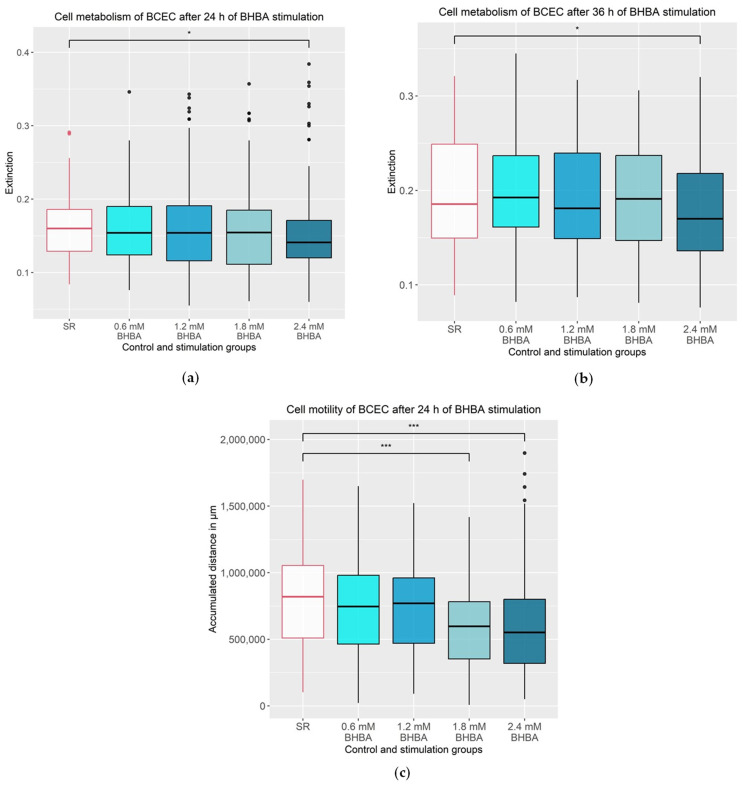
Cell metabolism and cell motility of BCEC after BHBA stimulation. (**a**) Cell metabolism was significantly reduced after 24 h stimulation with 2.4 mM of BHBA compared to the SR control group. The experiment was repeated 14 times and per group n = 177–199 extinctions were analysed; (**b**) cell metabolism was significantly reduced after 36 h stimulation with 2.4 mM of BHBA compared to SR control group. The experiment was repeated 14 times and per group n = 181–198 extinctions were analysed; (**c**) cell motility was significantly reduced after 24 h stimulation with 1.8 and 2.4 mM of BHBA compared to the SR control group. The experiment was repeated 4 times and per group n = 320 distances were analysed. (**a**–**c**): The data are presented as boxplots with the box representing the interquartile range. The interquartile range describes the range of values between the 75% and 25% quartile. The horizontal line indicates the median. The ends of the whiskers mark the minimum and maximum value. Outliers are shown with dots. The serum reduced control group (SR) is outlined in red. Statistical analysis was performed using Kruskall–Wallis test followed by the Dunn test with Bonferroni correction. The groups that differ significantly from the SR control group are marked with asterisks (* *p* ≤ 0.05 and *** *p* ≤ 0.001); BHBA: β-Hydroxybutyrate.

As illustrated in Figure 1a, the 24 h BHBA stimulation period affected the metabolism of the BCEC significantly. The metabolism was significantly reduced in the 2.4 mM BHBA stimulation group by an average of 5.82% compared to the control group (*p* = 0.044). Extension of the stimulation time up to 36 h significantly reduced cell metabolism by 8.47% in the 2.4 mM BHBA stimulation group compared to the control group (*p* = 0.024, Figure 1b).

As shown in Figure 1c, motility in BCEC was significantly reduced by 26.59% during 24 h of stimulation with 2.4 mM of BHBA (*p* < 0.001). The 24 h stimulation with 1.8 mM of BHBA also showed a significant decrease in cell motility by 26.85% (*p* < 0.001).

### 3.2. Effects of BHBA on the Gene Expression of IL6, RELA and TNF

**Figure 2:**
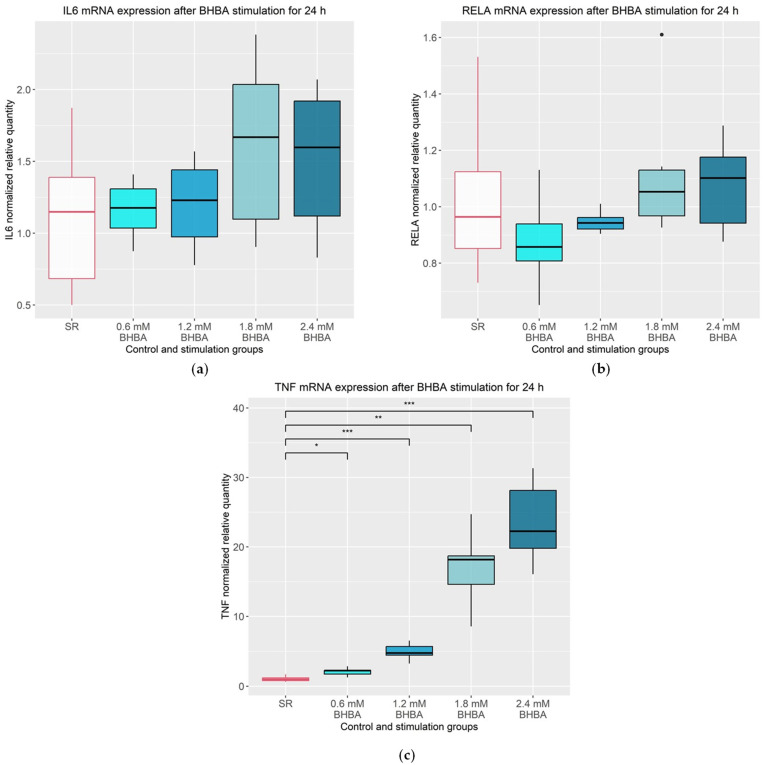
mRNA expression of *IL6*, *RELA* and *TNF.* (**a**) *IL6* mRNA expression was not altered after 24 h stimulation with 0.6, 1.2, 1.8 and 2.4 mM of BHBA compared to the SR control group. Statistical analysis was performed using ANOVA; (**b**) *RELA* (gene encoding for NF-κB p65) mRNA expression was not altered after 24 h stimulation with 0.6, 1.2, 1.8 and 2.4 mM of BHBA compared to the SR control group. Statistical analysis was performed using Kruskall–Wallis test; (**c**) *TNF* mRNA expression was significantly increased after 24 h stimulation with 0.6, 1.2, 1.8 and 2.4 mM BHBA compared to the SR control group. Serum reduced media (SR) served as control. Statistical analysis was performed using ANOVA followed by Games–Howell test. (**a**–**c**): The data are presented as boxplots with the box representing the interquartile range. The interquartile range describes the range of values between the 75% and 25% quartile. The horizontal line indicates the median. The ends of the whiskers mark the minimum and maximum value. Outliers are shown with dots. The experiment was repeated six times. The serum reduced control group (SR) is outlined in red. The groups that differ significantly from the SR control group are marked with asterisks (* *p* ≤ 0.05, ** *p* ≤ 0.01 and *** *p* ≤ 0.001); BHBA: β-Hydroxybutyrate.

The data of the mRNA expression of the cytokines are illustrated in Figure 2. The mRNA expressions of *IL6* and *RELA* were not altered by BHBA stimulation (Figure 2a,b). In contrast, *TNF* showed a significant increase in mRNA expression related to higher BHBA concentrations (Figure 2c). The experimental group stimulated with 0.6 mM of BHBA showed an increased *TNF* mRNA expression of 101% compared to the control group (*p* = 0.033). The stimulation with 1.2 mM of BHBA increased the mRNA expression by 382% (*p* ≤ 0.001), the stimulation with 1.8 mM of BHBA increased the mRNA expression by 1515% (*p* = 0.004) and the stimulation with 2.4 mM of BHBA increased the mRNA expression by 2188% (*p* ≤ 0.001), compared to the control group.

### 3.3. Effects of BHBA on the Gene Expression of PTGER2 and PTGES2

**Figure 3:**
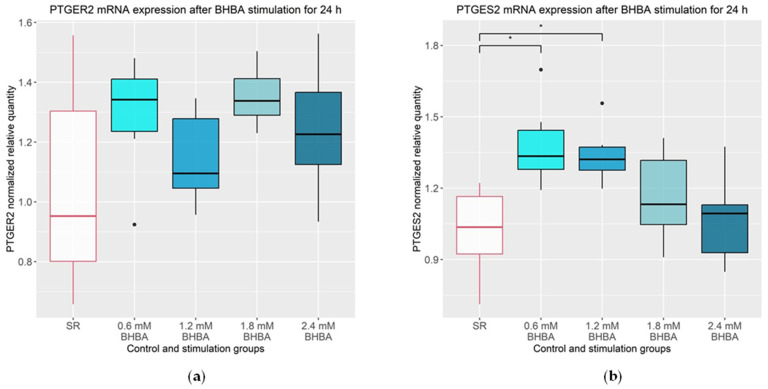
mRNA expression of PTGER2 and PTGES2. (**a**) *PTGER2* mRNA expression was not altered after 24 h stimulation with 0.6, 1.2, 1.8 and 2.4 mM of BHBA compared to the SR control group. Statistical analysis was performed using ANOVA; (**b**) *PTGES2* mRNA expression was significantly increased after 24 h stimulation with 0.6 and 1.2 mM of BHBA compared to SR control group. Statistical analysis was performed using ANOVA followed by Tukey’s HSD test. (**a**,**b**): The data are presented as boxplots with the box representing the interquartile range. The interquartile range describes the range of values between the 75% and 25% quartile. The horizontal line indicates the median. The ends of the whiskers mark the minimum and maximum value. Outliers are shown with dots. The experiment was repeated six times. The serum reduced control group (SR) is outlined in red. The groups that differ significantly from the SR control group are marked with asterisks (* *p* ≤ 0.05); BHBA: β-Hydroxybutyrate.

As shown in Figure 3a, *PTGER2* was not altered by BHBA stimulation. The *PTGES2* mRNA expression was significantly increased by 37% (*p* = 0.013) after stimulation with 0.6 mM of BHBA and by 34% (*p* = 0.032) after stimulation with 1.2 mM of BHBA, compared to the control group (Figure 3b).

### 3.4. Effects of BHBA on the Gene Expression of the Integrin Subunits ITGAV, ITGA6, ITGB1 and ITGB3

**Figure 4:**
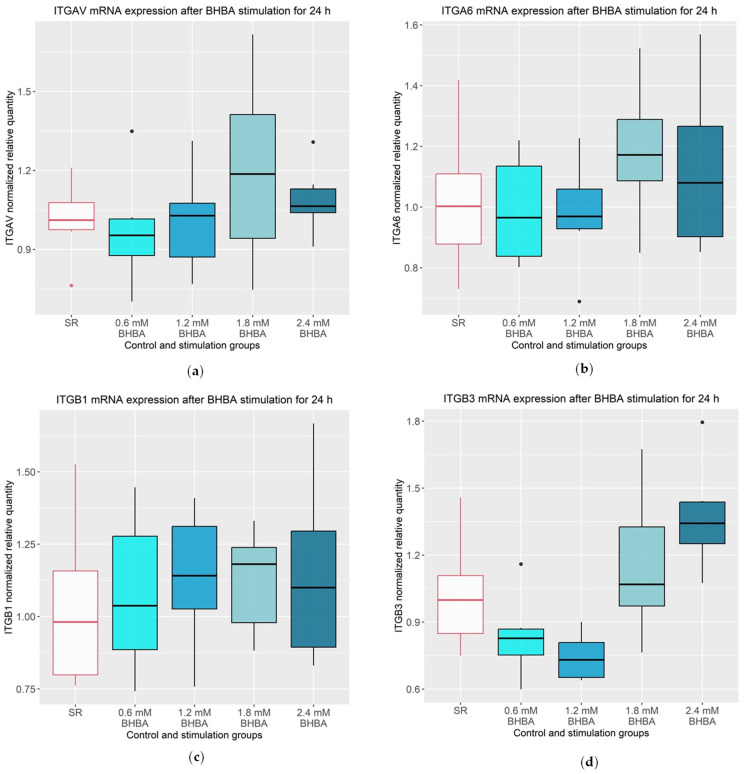
mRNA expression of integrin subunits *ITGAV*, *ITGA6*, *ITGB1* and *ITGB3*. (**a**) *ITGAV* mRNA expression was not altered after 24 h stimulation with 0.6, 1.2, 1.8 and 2.4 mM of BHBA compared to the SR control group; (**b**) *ITGA6* mRNA expression was not altered after 24 h stimulation with 0.6, 1.2, 1.8 and 2.4 mM of BHBA compared to the SR control group; (**c**) *ITGB1* mRNA expression was not altered after 24 h stimulation with 0.6, 1.2, 1.8 and 2.4 mM of BHBA compared to the SR control group; (**d**) *ITGB3* mRNA expression was not altered after 24 h stimulation with 0.6, 1.2, 1.8 and 2.4 mM of BHBA compared to the SR control group. (**a**–**d**): The data are presented as boxplots with the box representing the interquartile range. The interquartile range describes the range of values between the 75% and 25% quartile. The horizontal line indicates the median. The ends of the whiskers mark the minimum and maximum value. Outliers are shown with dots. The experiment was repeated six times. The serum reduced control group (SR) is outlined in red. Statistical analysis was performed using ANOVA. BHBA: β-Hydroxybutyrate.

The mRNA expression of the integrins is illustrated in Figure 4. The integrin subunits *ITGAV*, *ITGA6*, *ITGB1* and *ITGB3* did not show significant alterations following the stimulation with BHBA compared to the control group.

## 4. Discussion

To our knowledge, the effects of BHBA as a marker of ketosis on BCECs in vitro were investigated for the first time. In the present study, BHBA affected the cell viability, the gene expression of inflammatory markers and prostaglandin synthase, but it did not affect the integrin gene expression.

Dairy cows frequently suffer from pregnancy losses during early pregnancy [28]. Ketosis is one possible cause and is characterized by high BHBA blood levels [6,7,8]. In this context, the impact of high BHBA concentrations on fertility and especially on the function of placental cells has not yet been clarified. As mentioned before, a NEB with high levels of BHBA often occurs three weeks before and after calving [1]. And even if first insemination after calving takes place until well after that period, the damages in the endometrium caused by BHBA might nevertheless influence the establishment of a new pregnancy. There are studies about voluntary waiting periods up to 200 days, which might give the endometrium the possibility of recovering from these damages. However, very long voluntary waiting periods lead to extended lactation periods, which in turn lead to other problems like lower milk production, greater body condition score and an increased risk of metabolic disorders after calving [58].

In the present in vitro study, BCECs, the superficial epithelial layer of maternal placentomes, were stimulated with different BHBA concentrations (0.6, 1.2, 1.8 and 2.4 mM), according to the BHBA blood concentrations measured in affected animals [7,8,10,11,59,60]. The results indicate that high concentrations of BHBA (1.8 and 2.4 mM of BHBA for 24 and 36 h) affect cell metabolism and motility negatively. Comparable results have been reported in other studies [22,61,62,63,64,65,66,67]. The negative effect of BHBA on cell metabolism was primarily observed in cancerogenic cells, which are incapable of using BHBA as an energy source [61,62,63,68]. BHBA accumulates in these cells and inhibits the histone deacetylation, whereby pro-apoptotic and anti-proliferative genes are mainly coded [16,20]. In most non-cancerogenic cells, BHBA can be used as an alternative energy source, promoting proliferation and growth [20,69]. Although the BCEC of the present study are not cancerogenic, they may have transformed spontaneously during cultivation. Through a transformation, cells can change their properties and become, for instance, immortal. This has been described in other cell lines [70,71,72]. It is possible that cultivated BCECs are incapable of using BHBA as an energy source, as is observed in cancerogenic cells. However, a negative impact of BHBA and non-esterified free fatty acids (NEFA), a precursor of BHBA, on cell viability is also described in non-cancerogenic cells of the kidney, liver and endometrium [18,22,64,66,67,73]. In bovine hepatocytes and endometrial cells, cell viability was reduced by BHBA due to increased rates of apoptosis. The latter was induced by oxidative stress. Oxidative stress activates the NF-κB signalling pathway and induces an inflammatory response to BHBA, which includes high expression levels of cytokines like *TNF*, *IL6* and *IL1B* [22,23,66,74,75]. In the present study, BHBA likely induces an inflammatory response of the BCEC, since the cytokine *TNF* showed increased expression levels with rising BHBA concentrations. In contrast to the aforementioned studies, *IL6* and *RELA* (gene encoding for NF-κB p65) were not altered by BHBA stimulation in the BCEC. This might be explained by the fact that different cells were used for the experiments. Two of the previously mentioned studies used hepatocytes and not cells of the endometrium, and even the studies on bovine endometrial cells did not use exclusively caruncular cells of a pregnant cow like in the present study. It is well known that the immune response changes in pregnant cows, and therefore the origin of the BCEC from a pregnant animal, may also have led to the different results [36,76]. However, the assumption that BHBA induces an inflammatory response in the BCEC is supported by the ability of TNF to induce apoptosis in cells [77,78]. An increased apoptosis rate might explain the reduced cell metabolism of the BCEC.

Aside from the induction of apoptosis, increased *TNF* expression levels can have various effects on reproductive performance [79,80,81]. Studies on bovine embryos showed an inhibiting effect of TNF on blastocyst maturation in vitro [82]. In an in vivo study in cattle, a high dose of TNF directly infused into the blood circulation increased progesterone as well as the PGE2 concentration in the peripheral blood and resulted in an extended maintenance of the corpus luteum [83]. In other studies, TNF could induce the PGF2α production of stromal endometrial cells [84,85,86], and this might cause premature regression of the corpus luteum. Since the corpus luteum controls the oestrus cycle, increased *TNF* levels might lead to variable oestrus cycle intervals. Those findings and the reduction in cell metabolism and motility could explain the poor reproductive performance of dairy cows suffering from ketosis.

Like TNF, prostaglandins are mediators of the inflammatory response, but they are important regulators in the reproductive process as well [41,42,87]. In contrast to PGF2α, PGE2 promotes the maintenance of the corpus luteum [37,38,39]. In addition, PGE2 is important for the ovulation, hatching of the blastocyst and implantation [40,41]. Stimulation with 0.6 mM and 1.2 mM of BHBA increased the mRNA expression of *PTGES2* significantly compared to SR, but higher BHBA concentrations did not alter the *PTGES2* expression. BHBA did not affect the mRNA expression of *PTGER2* in the BCEC. A comparable in vitro study showed an effect of high concentrations of NEFA on prostaglandin synthesis in bovine endometrial cells with increased mRNA expression of *PTGES2* and *PTGER2* but with reduced PGE2 concentration in the cell supernatant. Negative feedback of PGE2 on the expression of its synthase and receptor is assumed. In contrast to the present study, a low concentration of NEFA had no effect [73]. This contradictory result might be due to the different stimulants. Unlike BHBA, fatty acids as part of the lipid metabolism affect prostaglandin synthesis by competitive inhibition of the synthesis of prostaglandins from arachidonic acid [88]. The fact that only low concentrations of BHBA affected the *PTGES2* expression does not indicate a negative impact of BHBA on the PGE2 synthesis. Blood concentrations of 0.6 mM of BHBA are physiological in cows and often show a positive effect, for instance, in the viability of bovine endometrial cells [11,13,22,60,66]. However, in some publications, blood concentrations of 1.2 mM of BHBA are the limit value of the diagnosis of ketosis in dairy cows [8,12], but the fact that higher BHBA concentrations did not affect *PTGES2* expression does not give any indications of a negative influence of BHBA on PGE2 synthesis. *TNF* mRNA expression equally increased at low BHBA concentrations but continued to increase at high BHBA concentrations.

The highest rates of pregnancy loss in high-yielding dairy cows are observed in early pregnancy [28]. During this time, crucial aspects are the maturation of the embryo and its implantation, whereby integrins play an important role [29,44,48]. In sheep, the integrin α_v_β_3_ is the binding partner of osteopontin during implantation and enables migration and adhesion of the trophoblast to the maternal luminal epithelium [44]. In the bovine endometrium, the expression patterns of integrins indicate a trophoblast giant cell migration mediated by the integrin α_6_β_1_ and the ligand laminin [48]. In the present study, stimulation with different concentrations of BHBA over 24 h did not alter mRNA expression of the integrin subunits *ITGAV*, *ITGA6*, *ITGB1* and *ITGB3*. Apart from the present study, there are only a few studies investigating the influence of BHBA or NEFA on integrin expression [89,90]. In mononuclear blood cells, the influence of high NEFA concentrations on gene expression was investigated using a transcriptome analysis in cows during early lactation. Interestingly, genes involved in cellular adhesion like integrins showed an altered expression. Animals with high NEFA blood concentrations showed an increased expression of the integrin subunit *ITGA4* but a decreased mRNA expression for integrin subunits *ITGAD* and *ITGA6* [89]. Besides the impact of integrins’ cell-to-cell binding on reproduction, they are also important for diapedesis of leukocytes and are thus essential for the immune defence against pathogens [89]. A study with human monocytes showed increased protein levels of the integrin lymphocyte function-associated antigen-1 after stimulation with BHBA concentrations between 0 and 10 mM in vitro. Therefore, it is assumed that hyperketonemia is involved in the devolvement of cardiovascular diseases [90]. To our knowledge, the present study is the first one that investigated the influence of BHBA on integrin expression in bovine endometrial cells. No significant effect of BHBA was observed. This might be due to methodical or biological reasons. The given experimental conditions might not be suitable, and the BHBA concentrations or the stimulation period could be modified. However, it is also possible that the expression pattern of the integrins was changed or that other integrins are affected by BHBA stimulation without showing changes in the expression level of the examined integrins itself. So far, it appears that the integrin mRNA expression of BCECs is not affected by BHBA.

## 5. Conclusions

The present study demonstrates that high concentrations of BHBA affect the physiology of BCECs by decreasing cell motility and metabolism. Additionally, an inflammatory response to BHBA stimulation was observed in the BCEC. The mRNA expression of *TNF* increased concomitantly with rising BHBA concentrations. An explanatory approach for these results is, on the one hand, an energy deficiency, since the BCEC possibly cannot use BHBA as an energy source, and on the other hand, the induction of an inflammatory process. Both might lead to higher apoptosis rates and negatively affect the functionality of maternal caruncles. This could lead to an impaired feto-maternal communication and explain the reduced reproductive performance during the peri-implantation period of ketotic cows. In addition, *PTGES2* expression was increased by stimulation with low BHBA concentrations, whereas high BHBA concentrations had no effect. The lowest BHBA concentration of 0.6 mM induced the strongest increase in *PTGES2* expression. This BHBA concentration is physiological, and therefore, this change is unlikely to have any negative effects.

## Figures and Tables

**Table 1:** Master mixes for RT-qPCR.

Master Mix	Mix 1 in µL	Mix 2 in µL	Mix 3 in µL
SYBR Green	10	10	10
Nuclease-free H_2_O	4	3	0
Primer forward	1.5	1.5	1.5
Primer reverse	0.5	1.5	4.5
cDNA (5 ng/µL)	4	4	4
Total	20	20	20

**Table 2:** Primers used in RT-qPCR.

Gene	Sequence (5′-3′) Forward Reverse	Mix	Product Length	Accession No.	References
*ACTB*	GATCAAGATCATCGCGCCCC ACAGTCCGCCTAGAAGCATT	1	160 bp	NM_173979.3	[56]
*GAPDH*	CAACATCAAGTGGGGTGATG GGCATTGCTGACAATCTTGA	2	202 bp	NM_001034034.2	[57]
*TNF*	GGTTCAAACACTCAGGTCCTCT CGGAGAGTTGATGTCGGCTA	2	79 bp	NM_173966.3	[56]
*IL6*	AAGCGCATGGTCGACAAAAT AAGCAAATCGCCTGATTGAACC	3	164 bp	NM_173923.2	[56]
*PTGES2*	CTATCTGGTGTCAGGGCAACC GGTGTACCAACCAGTCGTCC	2	212 bp	NM_001166554.1	PrimerBlast (NCBI)
*PTGER2*	CCTTGCCTTTCACGATTTTTGC CTCAGGATGGCAAAGACCCA	3	127 bp	NM_174588.2	PrimerBlast (NCBI)
*ITGA6*	TGCCACATATCACAAGGCTGA CTTACAGCGTGGTATCGGGG	2	151 bp	NM_001109981.2	PrimerBlast (NCBI)
*ITGAV*	AGCGCGTCTTCGATGTTTC TGTTGCCTGTGGCATCAAAC	3	145 bp	NM_174367.1	PrimerBlast (NCBI)
*ITGB3*	GAAGCAGAGTGTGTCACGGA ATGGGTCTTGGCATCAGTGG	2	142 bp	NM_001206490	PrimerBlast (NCBI)
*ITGB1*	TAGAGACTCCAGAGTGCCCC CCGTGTCCCATTTGGCATTC	3	180 bp	NM_174368.3	PrimerBlast (NCBI)
*RELA* (NF-kb p65)	TTTCAATGGACCCACCGACC TGATGGTGCTGAGAGATGGC	1	125 bp	NM_001080242.2	PrimerBlast (NCBI)

## Data Availability

The data presented in this study are available upon request from the corresponding author.
